# Evaluation of serum concentration of acute‐phase proteins (haptoglobin and serum amyloid A) in the affected Arabian foals with rhodococcosis

**DOI:** 10.1002/vms3.1005

**Published:** 2022-11-24

**Authors:** Ali Hassanpour, Sina Moghaddam

**Affiliations:** ^1^ Department of Clinical Science Tabriz Medical Sciences Branch Islamic Azad University Tabriz Iran; ^2^ Department of Internal Medicine Faculty of Veterinary Medicine University of Tehran Tehran Iran

**Keywords:** acute‐phase protein, Arabian foal, haptoglobin, *Rhodococcus equi*, serum amyloid A

## Abstract

**Background:**

Early detection of *Rhodococcus equi* pneumonia in foals is essential for horse health and for veterinarians.

**Objectives:**

This study aimed to demonstrate the usefulness of assessing the serum concentration of acute‐phase proteins (APPs) in the early diagnosis of pneumonia.

**Methods:**

The study evaluated APPs in 19 Arabian foals with *R. equi* pneumonia and compared them with 18 normal Arabian foals in equestrian clubs in Tabriz, Iran. Affected foals were identified through history, clinical findings and bacterial culture of tracheal washing. Biochemical methods and polymerase chain reaction tests were performed by examining the *16S rRNA* and *vapA* genes to confirm the diagnosis of bacterial isolates. Blood samples were taken from all sick and healthy horses, and their serum was isolated. APPs in the serum were measured in all the samples.

**Results:**

Rhodococcosis increased the serum concentration of haptoglobin (Hp) and serum amyloid A (SAA) (*p* < 0.001). The relationship between SAA and Hp was meaningful in the infected group (*r* = 0.933) but not in the healthy group. In cases where there are clinical findings of *R. equi* pneumonia, the concentration of SAA and Hp can help the effectiveness of treatment.‐

**Conclusions:**

Serum concentration analysis of APPs can be helpful in early diagnosis and successfully treating foals with *R. equi* pneumonia.

## Graphical abstract

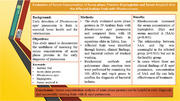

## 1 INTRODUCTION

Pets face various safety challenges throughout their lives. Depending on the species, sex, age and race, the first reaction to an external factor is activating specific immune responses (Belgrave et al., 2013). Rhodococcosis is a microbial disease caused by *Rhodococcus equi*. This bacterium is gram‐positive cocci and causes bronchopneumonia, pneumonia, pleura pneumonia, polyarthritis and osteomyelitis in foals. In adult mare, it may cause abortion. The disease is more prevalent among 1‐ to 4‐month‐old foals and could be chronic. Clinical signs of bronchopneumonia include cough, anorexia, fever, depression, tachypnea, wheezing and crackling in lung auscultation and seldom nasal discharge (Reed et al., 2017). Since the disease causes lung abscesses, it is possible not to hear abnormal sounds. It leads to non‐painful polyarthritis in 20% of infected foals and immune‐mediated uveitis in 10% of them. It may also form an abdominal cavity and other membrane abscesses. Ultrasound and radiography are effective in the diagnosis of lung abscesses. According to laboratory findings, leukocytosis and increased serum fibrinogen are observed in infected foals, and the serum amyloid A (SAA) level also rises in them (Cohen, 2006). Confirmation of the disease is based on the culture of tracheal secretion and bronchial isolation, which is obtained with the aspiration of the trachea or alveolar lavage. Culturing result is positive in 86% of cases. Polymerase chain reaction (PCR) is another definitive diagnosis that is 100% successful (Constable et al. 2017). This disease must be subtracted from interstitial pneumonia, viral pneumonia, bacterial pneumonia such as *Pasturella* and *Streptococcus* *pneumoniae* and parasitic pneumonia. Rhodococcosis causes many biochemical changes in serum levels, and the study of these changes will help prevent and control the disease; for example, checking the status of acute‐phase proteins (APPs) is important. APPs are some plasma proteins that increase or decrease during infection and inflammation. Most of the plasma proteins are synthesised in the liver, although some are synthesised in plasma cells like gama‐globulin or endothelial cells. In response to inflammation, these proteins increase (positive APPs) or decrease (negative APPs) plasma concentrations. Haptoglobin (Hp) and SAA are APPs that are important in horses and may increase at the inflammation time. SAA is one of the largest APPs in horses. The concentration of this protein is deficient during a healthy situation but increases rapidly in a few hours (100–1000 mg/L) in the inflammation phase (Browning et al., 2004; Chavan et al., 2005). The main APP in horses is SAA, although Hp is also one of the effective APPs (Cray et al., 2009). Other examples of different proteins in horses that can be measured include fibrinogen, albumin, Hp and α1‐glycoprotein. Free haemoglobins pass through the glomerulus and enter the tubules and are usually deposited there. However, the haemoglobin haptoglobin (Hb‐Hp) complex is a great complex that cannot pass the glomerulus. Thus, it seems that haemoglobin is responsible for preventing losses of free Hp from the kidney. This helps maintain haemoglobin's iron (Fe++) (Petersen et al., 2002; Pihl et al., 2013). In horses, studies have been performed on HP in viral diseases and after surgery, and some signs of increased serum HP have been observed in cases of nonseptic arthritis (Hultén et al., 2002; Kent & Goodal, 1991). The increase in plasma concentration SAA is proportional to the amount of damage to the tissue, and the serum SAA level increases within 6 h after inducing acute‐phase reaction (De Cozar et al., 2020; Satué et al., 2013). In toxins, SAA isotopes 1 and 2 are usually produced in the liver and secreted into the bloodstream after activating acute‐phase reaction. Isotope 3 is found in liver cells, several different tissues, including endothelial lining in the lungs and gastrointestinal tract, and inflamed synovial fluid in horses during acute‐phase reaction (Berg et al., 2011; Satué et al., 2013).

This study examined the serum levels of APPs (Hp and SAA) in foals infected with *R. equi* and compared them with those in healthy foals.

## 2 MATERIALS AND METHODS

This study was conducted on 19 infected Arabian foals in Tabriz, Iran, for 3 months. The infection of the foals was confirmed according to clinical signs and laboratory findings.

### 2.1 Tracheal washing

After prescribing a sedative (xylazine 0.5 mg/kg IV), the middle third of the foal's neck was shaved. Next, 2 to 5 ml lidocaine was injected subcutaneously between two tracheal rings aseptically. A small incision (0.5 cm) was then made in the area, and a cannula/needle was inserted at the incision point. Afterwards, 50 ml of 0.9% normal saline was introduced into the trachea with a special catheter, and then aspiration started.

### 2.2 Bacterial culture

Tracheal aspiration samples were transferred to a diagnostic laboratory inside a special tube. In the laboratory, the samples were cultured on a blood agar medium containing 5% sheep blood and incubated at 37°C for 48 h under aerobic conditions. Mucoid colonies, which are smooth, shiny and non‐hemolytic, represented *R. equi* bacteria, and biochemical and Christie Atkins Munch Peterson (CAMP) tests were used to confirm the diagnosis.

### 2.3 Preparation of DNA from cultured colonies

The isolates were stored in a bacterial laboratory after biochemical approval. For short‐term storage for daily experiments, Infusion Agar was cultured linearly and stored in the refrigerator. Glycerin was stored at −70°C for long‐term storage to prevent genetic changes. Pure and isolated biochemical isolates were used for DNA extraction. To this end, the gram‐positive bacterial DNA extraction kit of Tissue Genomic DNA Extraction Mini Kit (FavorPrep, Cat No. FATGK001) was used. Next, a complete loop was transferred from the bacterial colony to a microtube and centrifuged at 13,000 rpm. The bacterial pellet was dissolved in 50 μl of lysozyme reaction solution, vortexed for a few seconds and incubated at 37°C for 60 min. In the next phase, 20 μl of proteinase K along with 200 μl of FATG2 buffer was added to the sample. Subsequently, the mixture was vertexed, incubated at 60°C for 30 min and then vortexed occasionally during incubation. Next, 200‐μl ethanol 96% was added to the sample mixture. An FATG Mini Column was placed in a collection tube. The mixture was then carefully transferred to the column, centrifuged at 13,000 rpm for 1 min and placed in a new collection tube. Afterwards, 400‐μl wash buffer 1 was added to the column, which was then centrifuged at full speed for 1 min and discarded flow‐through. Next, 750‐μl wash buffer 2 was added to the column, which was then centrifuged at full speed for 1 min and discarded flow‐through. The column was centrifuged at full speed to dry for an additional 3 min. Subsequently, 100 μl of preheated elution buffer was added to the membrane of the FATG Mini Column, and the column was centrifuged at full speed for 2 min to elute DNA. The final solution was analysed for DNA quality and quantity using nanodrop and agarose gel. The DNAs were stored at −20°C for further research. After extraction, all the DNAs were concentrated using a photometer (BioRad), and their values were checked at optical density (OD) 260/280 and OD 280/230, which were suitable in terms of purity. Also, 5 μl of each DNA were taken on an agarose gel to be tested for quality, which yielded good results.

### 2.4 Molecular testing

All the PCR samples were tested for the presence of *16S ribosomal subunit* and *vapA* genes to confirm the presence of *R. equi* DNA. Nucleotide sequences of primers include 16S forward, TCG TCC GTG AAA ACT TGG G; 16S reverse, CGA CCA CAA GGG GGC CGT; VP forward, GGT TCT CGT AAC GCT ACA ATC and VP reverse, GGT TCG TCT TTC TGA AGG TT (Sellon et al., 2001). Each PCR mixture contained 5 μl of DNA, 5 pmol of each primer and 2.5 U of PCR Master Mix 2x (Cat No. MM2011, Sinacolon), making up a total volume of 25 μl. The first series of PCR reactions involved DNA denaturation at 94°C for 2 min followed by 35 amplification cycles, each comprising of denaturation at 94°C for 35 s, primer annealing at 64°C for 30 s and extension at 72°C for 1 min. Each test run included positive (*R. equi* strain microbial collection of Mr. Sharghi Bacterial Laboratory) and negative controls. Finally, 1.5% agarose gel was stained with DNA Safe Stain (cat No. EP5082, Sinaclon) and electrophoresed with 85 volts for 90 min in 1X Tris Borate EDTA (TBE) buffer to read the PCR results.

### 2.5 APP monitoring

A 20‐ml blood sample was collected from the jugular vein of each diseased foal and frozen after the serum was separated from each sample. After separating serum, levels of APPs (Hp and SAA) were measured. Serum Hp was measured using a specific enzyme linked immunosorbent assay (ELISA) kit (K‐ASSAY Horse Hp ELISA, Kamiya Biomedical Company). This kit is specific for measuring horse serum Hp and works based on an immunoassay test. After separating serum or plasma, they were diluted and incubated for 45 min in the ELISA microtubes. The streptavidin horseradish peroxidase (HRP) reagent was added to the samples, and the samples were incubated for 30 min. This enzyme reagent is connected to anti‐Hp antibodies. The serum Hp of the samples reacted with anti‐Hp antibodies. These enzymes were labelled previously banded serum Hp antibodies. After washing, the antibody‐banded enzyme was measured by adding tetra methyle benzydine (TMB) with a 450‐nm wavelength. The level of these enzymes shows the exact serum Hp level (Eckersal, 2000).

SAA was measured using the ELISA kit (Innovative Research) based on an immunoassay test. SAA reacted with SAA antibodies. After separating nonbonded proteins with washing, enzyme‐conjugated antibodies and the HRP reagent were detected. These enzymes were labelled as antibodies banded with SAA. After washing, the antibody‐banded enzyme was measured by adding TMB with a 450‐nm wavelength. The level of these enzymes shows the exact SAA level.

### 2.6 Statistical analysis

Twenty samples of *R. equi* infected foals and 20 healthy foals were selected based on the $n=\frac{z^{2}\left(1-\frac{\alpha }{2}\right)\times \sigma^{2}}{d^{2}}$ formula. The SPSS software version 26 was used to compare parameters between the two groups and determine the relationship between serum parameters. Correlation statistical methods were used in the treatment group. A significance level of *p* < 0.05 was used for analyses. The final number of samples was 19 foals in the treatment group and 18 Arabian foals in the control group due to sample loss resulting from lyse of serum

## 3 RESULTS

**FIGURE 1:**
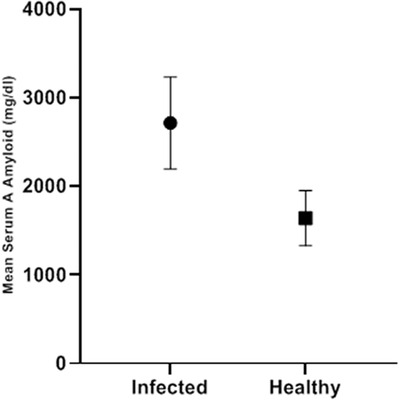
Mean serum amyloid A (SAA) levels in infected and control groups

The mean SAA level was 2715.24 ± 516.6 mg/dl in the infected foals and 1640.21 ± 312.49 mg/dl in the controls. Mean SAA levels showed significant differences between the two groups (*p* < 0.001). The highest and lowest levels were 3467.8 and 1950.5 in the infected group and 2260.3 and 1116.9 in the control group, respectively (Table 1 and Figure 1).

**TABLE 1:** Comparison of serum levels of acute‐phase proteins (haptoglobin [Hp] and serum amyloid A) between Arabian foals with *Rhodococcus equi* infection and control group (at *p* < 0.05 meaningful)

Serum parameter	Group	Number	Mean ± SD	T	*p*‐value
Amyloid A (mg/dl)	Infected	19	2715.24± 516.60	7.70	< 0.001
	Healthy	18	1640.21 ± 312.49		
Hp (mg/dl)	Infected	19	1321.36 ± 204.23	10.72	< 0.001
	Healthy	18	684.23 ± 154.96		

**FIGURE 2:**
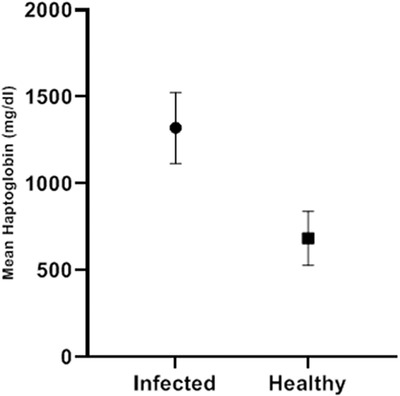
Mean serum haptoglobin levels in both groups

The mean serum Hp level was 1321.36 ± 204.23 mg/dl in the *R. equi* infected foals and 684.23 ± 154.96 mg/dl in the control group, and the mean difference between the two groups was significant (*p* < 0.001). In the infected group, the highest level was 1654.6 and the lowest level was 935.4, while in the control group, these values were recorded at 430.7 and 943.4, respectively (Table 1 and Figure 2).

The relationship between SAA and Hp was meaningful (*r* = 0.933) in the infected group but not significant in the control group (Table 2).

**TABLE 2:** Comparison of correlations between Hp and SAA in the patient group (at *p* < 0.05 meaningful)

Group	Correlation coefficient (*r*)	*p*‐value
Infected	0.933	< 0.001
Healthy	−0.033	0.896

## 4 DISCUSSION

*Rhodococcus equi* causes mild to severe respiratory clinical signs in foals. (Reed et al., 2017). Infection from *R. equi* in foals has been reported in the United States (Muscatello et al., 2007). In a study, plasmid virulence genes of *R. equi* and its virulence showed that this bacterium could survive a long time in the environment and live tissues (Letek et al., 2008). Research has found that age, immune suppression and environmental condition have the highest effect as risk factors (Chaffin et al., 2000). It is an inflammatory process that can cause changes in serum levels of APPs and inflammatory markers.

In foals infected with *R. equi*, the measurement of SAA was expressed as a diagnostic criterion (Cohen et al., 2005). SAA was measured in an infected horse with influenza and reported as an AAP and inflammatory marker (Hultén, Sandgren, et al., 1999). In another study, amyloid A and Hp in serum and peritoneal fluid of horses with colic were measured, indicating that SAA was 0.05 mg/L, and changes in serum Hp and SAA levels were associated with changes in the peritoneal fluid (Pihl et al., 2013). In diseases such as mastitis, metritis and retained placenta, Skinner et al. have expressed increases in serum Hp; also, the elevation of serum Hp in milk fever has been reported (Skinner et al., 1991). In some studies, serum Hp is used to predict the disease. Carter et al. noted using Vitamin E and its effect on serum Hp levels in sick animals with a respiratory disorder (Carter et al., 1962). In the present study, the SAA and Hp concentrations significantly increased for foals with *R. equi* due to inflammatory conditions.

A study was performed on a relatively large population of involved foals and the control group on similar farms. In the study, SAA concentrations were evaluated in 212 horses, and no predictive value was found at the onset of clinical signs of pneumonia (Cohen et al., 2005). Also, in a study with a smaller population, screening with SAA did not help detect pneumonia caused by *R. equi* in an involved farm. Only two of the six cases with pneumonia had elevated serum SAA concentrations (Passamonti et al., 2015). Contrary to the above studies, the present study showed that pneumonia caused by *R. equi* could increase the APPs. SAA is usually rapidly increased due to tissue damage, inflammation or infection, although it can be detected normally in healthy horses (Hultén, Tulamo, et al., 1999). Serum SAA concentrations in foals have been reported to be higher than normal during infection with *R. equi* or other bacterial infections (Hultén & Demmers, 2002). Also, the serum SAA concentration in horses was directly related to the clinical signs of respiratory diseases and increased (Hultén et al., 1999). Along with studies mentioned in the present study, an increase in serum SAA was observed following rhodococcosis. One study found that measuring SAA was as useful as measuring fibrinogen, and it was better than predicting which horse was causing the clinical signs of *R. equi* pneumonia. The SAA concentration in this study was significantly higher in pneumonic foals than in healthy foals (Giguère et al., 2016). Hp usually rises 24 h after inflammation and can be a good diagnostic tool for chronic inflammatory disease (Cray & Belgrave, 2014). In the present study, the amount of Hp increased, compared to the control group, due to the incidence of pneumonia caused by *R. equi*. This study clarified some studies suggesting that the concentrations of SAA and Hp were the same between horses with bacterial infection *R. equi* and clinically healthy foals. The reason is that the concentration of SAA and Hp was significantly higher in foals with *R. equi* due to inflammation and pulmonary involvement than in clinically healthy foals. SAA has a relatively short half‐life, making it an ideal marker for the early stages of inflammation and monitoring the therapeutic response. Therefore, a one‐time analysis of SAA concentrations identifies the possibility of inflammation or infection in the body. Serial analysis during the treatment of foals can be helpful for foals with *R. equi*. Differences between studies can be explained by size differences in the studied populations.

## 5 CONCLUSION

The findings of this study indicate that *R. equi* causes increase in the serum level of APPs, Hp and amyloid A, and increasing one serum parameter depends on and drives improvement in the other one. Therefore, these cases can be considered in treating and controlling the disease. Of course, serial sampling studies are needed to measure these serum parameters. It is also suggested to use additional methods for early detection of pneumonia in foals.

## CONFLICTS OF INTEREST

The authors declare that there is no conflict of interest.

## AUTHOR CONTRIBUTIONS

The study was designed by Ali Hassanpour and Sina Moghaddam. Laboratory work was performed by Sina Moghaddam. All authors contributed to data analysis and interpretation. Ali Hassanpour prepared the initial manuscript draft, and all authors contributed to the manuscript revision and approved the final version.

### PEER REVIEW

No, I would not like my name to appear with my report on Publons https://publons.com/publon/10.1002/vms3.1005

## FUNDING INFORMATION

The authors received no specific funding for this work.

## ETHICS STATEMENT

The authors confirm that the ethical policies of the journal, as noted on the journal's author guidelines page, have been adhered to and the appropriate ethical review committee approval has been received. Animal experiments were carried out by the guidelines described by the Institutional Animal Care and Use Committee (IACUC).

## Data Availability

The data that support the findings of this study are available from the corresponding author upon reasonable request.
